# Role of B-Cell in the Pathogenesis of Systemic Sclerosis

**DOI:** 10.3389/fimmu.2022.933468

**Published:** 2022-07-12

**Authors:** Benjamin Thoreau, Benjamin Chaigne, Luc Mouthon

**Affiliations:** ^1^Department of Internal Medicine, National Referral Center for Rare Systemic Autoimmune Diseases, Cochin Hospital, AP‐HP, CEDEX 14, Paris, France; ^2^Université Paris Cité, Paris, France; ^3^INSERM U1016, Cochin Institute, CNRS UMR 8104, Université Paris Cité, Paris, France

**Keywords:** systemic sclerosis, B-cell, pathogenesis, B-cell receptor (BCR), interleukine 6 (IL-6), autoantibodies, transcriptomic, rituximab

## Abstract

Systemic sclerosis (SSc) is a rare multisystem autoimmune disease, characterized by fibrosis, vasculopathy, and autoimmunity. Recent advances have highlighted the significant implications of B-cells in SSc. B-cells are present in affected organs, their subpopulations are disrupted, and they display an activated phenotype, and the regulatory capacities of B-cells are impaired, as illustrated by the decrease in the IL-10+ producing B-cell subpopulation or the inhibitory membrane co-receptor density. Recent multi-omics evidence highlights the role of B-cells mainly in the early stage of SSc and preferentially during severe organ involvement. This dysregulated homeostasis partly explains the synthesis of anti-endothelial cell autoantibodies (AECAs) or anti-fibroblast autoantibodies (AFAs), proinflammatory or profibrotic cytokines (interleukin-6 and transforming growth factor-β) produced by B and plasma cells. That is associated with cell-to-cell interactions with endothelial cells, fibroblasts, vascular smooth muscle cells, and other immune cells, altogether leading to cell activation and proliferation, cell resistance to apoptosis, the impairment of regulatory mechanisms, and causing fibrosis of several organs encountered in the SSc. Finally, alongside these exploratory data, treatments targeting B-cells, through their depletion by cytotoxicity (anti-CD20 monoclonal antibody), or the cytokines produced by the B-cell, or their costimulation molecules, seem interesting, probably in certain profiles of early patients with severe organic damage.

## Highlights

- B-cells are key players in SSc, implicated in fibrosis and autoimmunity
- B-cell subsets are disrupted
- B-cells are activated and lose their regulation properties
- B-cells produce autoantibodies and cytokines
- B-cells crosstalk with numerous cells including fibroblasts, endothelial cells, T cells, dendritic cells, and other immune cells
- Treatments targeting B-cells, through their depletion by cytotoxicity, or the cytokines produced by the B-cell, or their costimulation molecules seem interesting, probably in certain profiles of early patients with severe organic damage

## 1 Introduction

Systemic sclerosis (SSc) is a rare multisystem autoimmune disease of unknown cause characterized by fibrosis, vasculopathy, and autoimmune features (1, 2). SSc is responsible for visceral manifestations such as interstitial lung disease (ILD) and pulmonary arterial hypertension (PAH), which represent the two main causes of death (3). SSc is also responsible for significant disability, handicap, and a hampered quality of life.

The pathogenesis of SSc is not fully understood. Still, significant progress has been made in the last 20 years in the understanding of the mechanisms contributing to the occurrence of vasculopathy and fibrosis. In patients with SSc, the vascular tone is dysregulated, leading to perturbed interactions between endothelial cells, vascular smooth muscle cells (VSMC), and extra-cellular matrix (ECM) components participating in vascular remodeling and occlusion. ECM accumulation is mostly the consequence of fibroblast activation (4). Since skin fibrosis is the main hallmark of this disease, research initially focused on the fibroblast. However, the demonstration in the early state of the disease of cutaneous lymphocyte infiltration before any process of fibrosis (5), the presence of auto-antibodies, imbalances in the B-cell subsets, and a hyperactivated state repositions the B-cell as an actor in the pathogenesis of SSc.

We herein provide an overview of B-cells in the pathogenesis of SSc from human data and animal models. We review B-cell distribution, B-cell dysfunction, and B-cell participation in inflammatory and fibrotic lesions, with emphasis on recently published data and their implication for patient prognosis. Lastly, we discuss the different therapeutic options targeting B-cells or pathways involved in B-cell activation.

## 2 B-cells Are Found in Affected Organs

**Figure 1:**
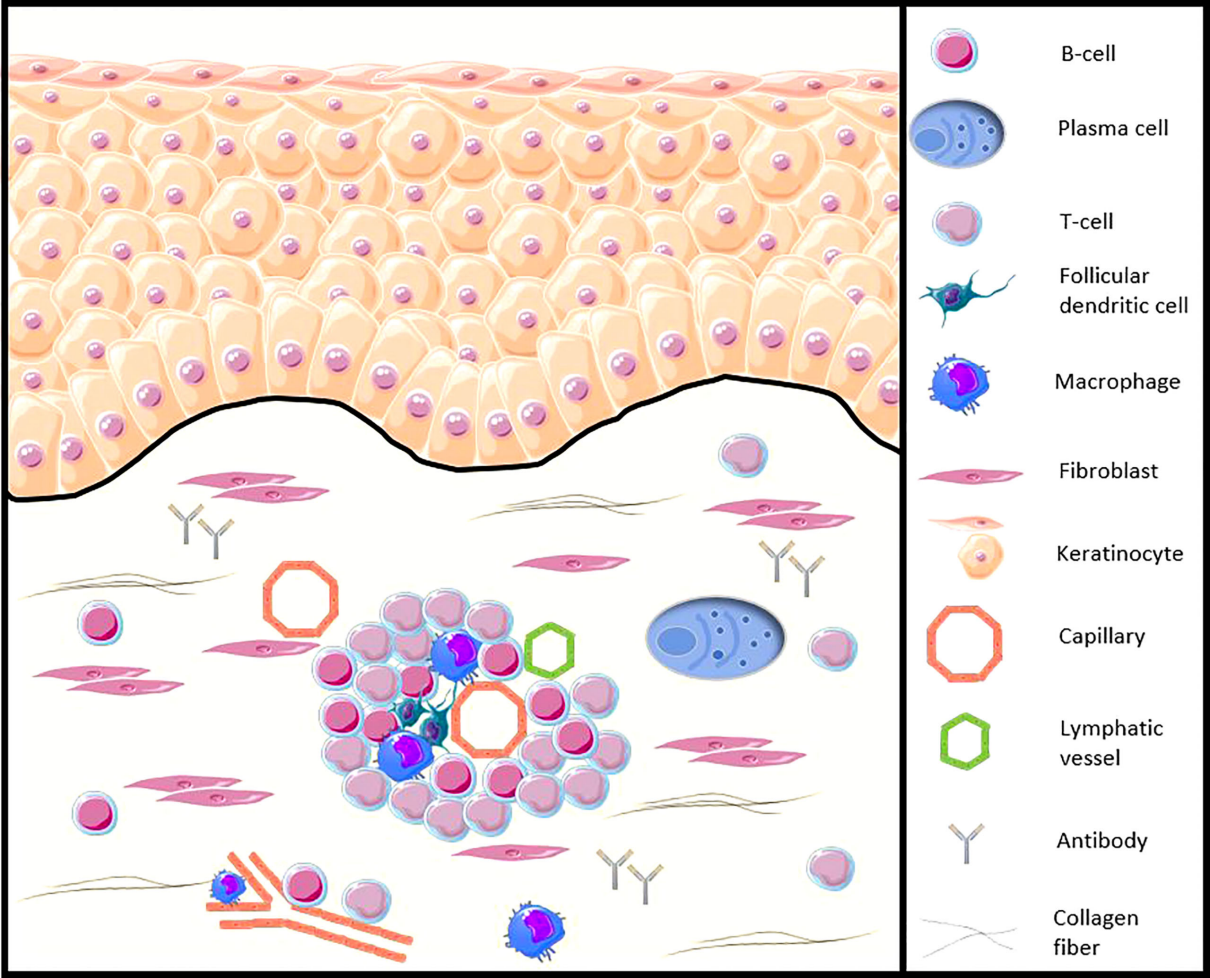
Illustration of the B-cell presence in the SSc patients skin (adapted from Fetter et al. Cells, 2020 [13)]. CD20^+^ B-cells and plasma cells infiltrate the skin of SSc patients, from the early stages of the disease, especially around blood vessels, accompanied by other peripheral blood mononuclear cells, such as CD3^+^ T cells or follicular dendritic cells. B-cells participate in the functions of antigen-presenting cells, production of autoantibodies and proinflammatory and profibrotic cytokines, as well as direct contact with other cells such as the fibroblast.

Early presence of various innate or adaptative immune cells infiltrates which B and plasma cells have been demonstrated in the skin, lung, or gastrointestinal tract of SSc patients (6–10). Recent genetic and immunohistochemical data reinforce the implication of B-cells in the skin of SSc patients, from the early stages of the disease, in particular with the demonstration of the presence of CD20^+^ cells in the skin of patients and an activated B-cell genetic signature (11, 12) and illustrated in **Figure 1**. Moreover, in SSc patients, the presence of CD20^+^ B-cells in clinically affected skin seems to be greater compared to unaffected skin. Based on immunohistochemical data, the presence of inflammatory cells, including B-cells, in cutaneous samples from areas clinically involved and uninvolved by fibrosis may suggest the possibility of detecting pathological changes before the clinical onset of skin fibrosis (12).

Concerning the lung, a higher percentage of CD19^+^ B-cells in bronchoalveolar lavage fluid was found in SSc patients with ILD and could be correlated with worsening of the DLCO during pulmonary function tests (14). The occurrence of patchy lymphocytes and plasma cells or real lymphoid follicle organization in the lung examination of SSc-related ILD or PAH patients has also been described (15, 16).

## 3 B-cells in SSc Exhibit an Activated Phenotype in Some Unbalanced Subsets

### 3.1 Distribution of B-Cell Subsets in SSc

Peripheral CD3^-^ CD19^+^ B-cells represent around 10% of total white blood cells (WBCs) and the last 30 years allowed us to isolate several peripheral B-cell subsets with specific phenotypes, including naïve, marginal zone, switched memory B-cells, and plasma cells (17). In SSc patients, the frequency of B-cells (relative count) among peripheral blood mononuclear cells is increased compared to healthy controls (18, 19). Contradictorily, data vary dramatically in the absolute count of peripheral B-cells in SSc patients according to the authors, reported to be increased (18, 20) similar (21, 22), or decreased (23) compared to healthy controls. These differences could be explained by differences in the methods used, the threshold definitions, and/or the use of glucocorticoids and/or immunosuppressant drugs. Thus, B-cell subsets in SSc patients are unbalanced, with an expansion of naive and transitional B-cells with a decrease in memory B-cells, switched memory B-cells, and IL-10^+^-producing regulatory B-cells (Bregs), particularly in steroid-untreated patients (16, 18–20, 22, 24–27), with a higher circulating plasma cell molecular signature (28). Transitional B-cells represent a maturing subset undergoing peripheral selection, whereas switched memory B-cells are long-lived cells persisting in a quiescent state (17). These immune dysregulations lead to inadequate B-cell homeostasis and favor the emergence of autoimmune reactions.

### 3.2 B-Cells Overexpress Activated Phenotype in SSc

In SSc patients, the transitional and memory B-cells are constantly activated compared with controls. The expression levels of the co-stimulatory molecules CD80, CD86, and CD95 are increased, with an increased proportion of IL-6^+^-and IL-6 receptor^+^ (IL6R) B-cells (4, 18–20). The overexpression of CD69, CD5, CD86, CD95 markers, and the IL6R is associated with the diffuse cutaneous SSc (dcSSc) form and the early stage of disease, suggesting the implication of the activated B-cells in the active and aggressive forms of SSc (16). The decrease in the memory B-cell compartment found in SSc compared to healthy controls is probably due to increased apoptosis (18). B-cells are not only a cellular precursor of differentiated secreting-antibody cells. The B-cells are involved in various essential regulatory immune processes like the antigen presentation, T-cell maturation and differentiation, the organization of the architecture of the lymphoid organ, and the cytokine synthesis (29). The upregulation of the CD80/86–CD28 costimulatory molecules contributes to activating autoreactive T cells and increasing the secretion of profibrotic cytokines in SSc (30). Simultaneously, the ability of B-cells to produce IL-10 is altered, participating in downregulating Breg (20, 22). Additionally, B-cells from SSc patients express increased programmed death-1 (PD-1) and PD-1 ligand 2 (PD-L2), a co-stimulatory CD28/B7 family system, which contributes to the impairment of immune tolerance (31). This disrupted homeostasis of peripheral B-cell subsets in SSc could be secondary to an overexpression of activation markers in certain subsets, counterbalanced by increased CD95-mediated apoptosis in memory B-cells (18). This disrupted homeostasis encountered in B-cells in SSc patients probably influences many of their regulatory immune functions.

## 4 B-cell Receptor, Intracellular Signaling and Intrinsic Pathways of B-cells Are Disrupted in SSc

### 4.1 B-Cell Receptor and Its Co-Receptors

**Figure 2:**
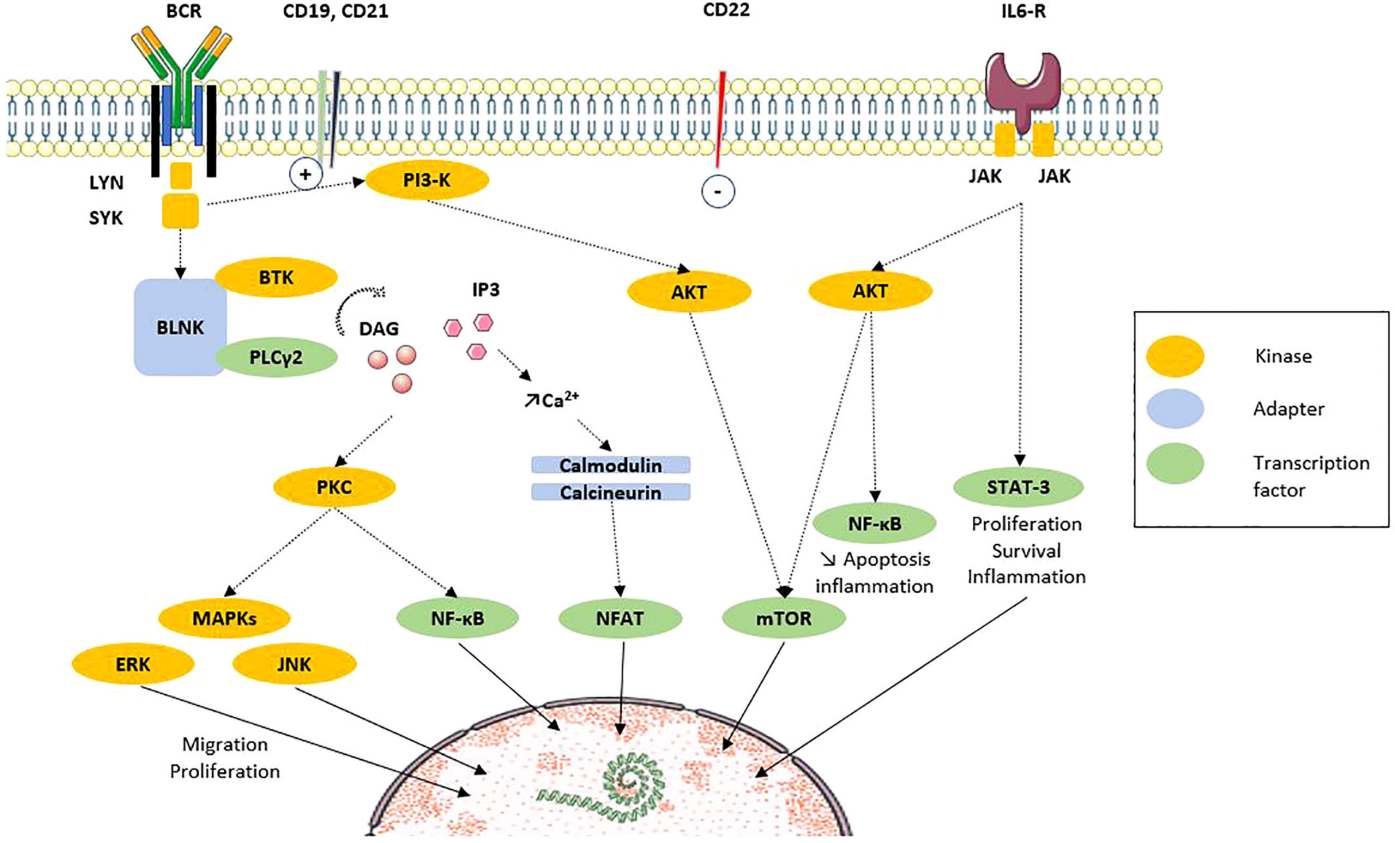
B-cell signaling *via* BCR and costimulation molecules (adapted from Dal Porto et al. Mol Immunol, 2004 [32)]. After ligation of antigen on the B-cell antigen receptor (BCR) at the membrane, signals are transduced and propagated by several protein phosphorylations, modifications and interactions. The end point is the regulation of transcription factors that regulate the expression of genes. Recent results highlight the participation of intracellular signaling perturbations in B-cell including defective phosphorylation of MAPK, STAT3 or mTOR molecules. Solid line: translocation. Dotted line: direct action. AKT, protein kinase B; BCR, B-cell receptor; BLNK, B-cell linker protein; BTK, Bruton’s tyrosine kinase; CD, cluster of differentiation; DAG, diacylglycerol; ERK, extracellular signal-regulated kinases; IL, interleukin; IL6-R, IL6 receptor; IP3, inositol triphosphate; JAK, Janus kinases; JNK, c-Jun N-terminal kinase; Lyn, Lck/Yes novel tyrosine kinase; MAPK, mitogen associated protein kinase; mTOR, mammalian target of rapamycin; NFAT, Nuclear factor of activated T cells; PI3K, phosphoinositide 3-kinase; PLCγ, phospholipase Cγ2; PKC, protein kinase C; STAT3, signal transducer and activator of transcription 3; SYK, spleen tyrosine kinase.

The activation of B-cells mediated by antigen implicates the B-cell receptor (BCR), many co-receptors, and coactivation molecules (**Figure 2**). Several components of this axis are altered during the SSc. In SSc patients, the BCR diversity is altered, which is related to the alteration of B-cell development. In particular, the VDJ rearrangement is altered, leading to an excess of membrane IgD diversity and a defective mutation load identified as a SSc-related PAH signature (33). In humans, the membrane density of the CD19 co-receptor at the membrane of peripheral B-cells in SSc patients was rated 20% higher than that in healthy controls, which seems to be specific to SSc since it is not the case in SLE (18, 21). Additionally, the expression of CD21, another positive regulator of the BCR signal, is also higher at around 20% (18, 21). Several membrane inhibitors, such as CD22 or CD35, have been found decreased on B-cells, close to a factor of 2 for CD35, especially on memory B-cells in SSc patients with peripheral vascular modified Medsger scale altered (≥1), with diffuse cutaneous SSc (dcSSc), and ILD (19, 34).

### 4.2 B-Cell Activating Factors

Independently of antigen-mediated mechanisms, BAFF and APRIL pathways are implicated in the pathogenesis of SSc. B-cell activating factor (BAFF) and APRIL pathways, both members of the tumor necrosis factor (TNF) superfamily, play a critical role in the activation, survival, and maturation of B-cells, and are involved in many disorders like transplantation/graft versus host diseases (GVHD) and autoimmune diseases (35).

First, in the type 1 tight skin mouse model (TSK/+), an increase in serum BAFF levels has been reported. Its blockage inhibits the development of skin fibrosis and autoantibody production or TGF-β and IL6 mRNA levels in the skin (36). Thus, in humans, serum BAFF and APRIL levels are higher in SSc patients compared to healthy controls by about 25 to 50% for BAFF (22, 37–39), as well as the amount of one of its specific receptors (BAFFR) at the surface of B-cells (37). Positive correlations between serum levels of BAFF and APRIL and modified Rodnan skin score (mRSS), early dcSSc, ILD and IL-6, serum IgG level, and anti-Topo I Ab titers are reported (22, 37–42).

### 4.3 Intracellular Signaling and Intrinsic Pathways of B-Cells Are Upregulated in SSc

Beyond alterations targeting extrinsic activators, defects in intracellular B-cell signaling, mainly tyrosine kinases, have been documented in many autoimmune diseases, including SLE, rheumatoid arthritis (RA), and SSc. The involvement of calcium storage has also been reported.

In TSK/+ B-cells, the CD19-mediated intracellular response to stimulation appears to be exacerbated, with an increase in intracellular calcium [Ca^2+^]i storage (43, 44). Then, spleen tyrosine kinase (SYK) has been reported as hyperphosphorylated in B-cells of murine sclerodermatous chronic graft-versus-host disease (Scl-cGVHD) (45). Experimental use of fostamatinib, an inhibitor of SYK in Scl-cGVHD or BLM models, appears to contribute to decreasing phospho-SYK, TGF-β expression, and tissue fibrosis (45, 46).

Thus, Lyn kinase, the first signal transducing kinase involved after BCR activation, is downregulated in CD22^low^ B-cells from SSc patients (34). Moreover, after activation, defective phosphorylation of downstream MAPK, STAT3 (20, 25) or mammalian target of rapamycin (mTOR) (22) has been reported, especially in CD27+ transitional or memory B-cells by some authors (20, 25).

### 4.4 Genomic, Transcriptomic, and Proteomic Data Argue a B-Cell Signature in SSc

Large-cohort genomic studies have identified associations between genes involved in B-cell signaling and the SSc phenotype, including B-cell scaffold protein with ankyrin repeats 1 (BANK1), B lymphoid kinase protein (BLK), c-src tyrosine kinase (CSK) or intracellular protein tyrosine phosphatase (PTNP22) genes (47).

All studies on global gene expression in peripheral blood cells from SSc patients revealed the interferon IFN-inducible signature (48). Moreover, the profiles show a heterogeneity of the signatures according to certain clinical characteristics. The presence of an anti-topoisomerase or anti-U1 RNP antibody is associated with a high IFN-inducible signature, in contrast to the presence of an anti-centromere antibody or digital ulcer (49, 50).

Transcriptomic analysis of skin biopsies of SSc patients has found a B-cell signature in 69% of cases (51), with an increased expression of B-cell-related genes like genes related to light and heavy chains of immunoglobulins, with genes that contribute to increased extracellular matrix synthesis or activation of T cells and correlated with skin thickness. These results are confirmed by immunohistochemistry staining on skin biopsy (11, 51). Whole skin transcriptomic analysis of SSc patients also showed discrimination according to the profiles of antinuclear antibodies (ANA) (52). Interestingly, adaptive immune signature, especially B-cell signature in the skin, was associated with early and diffuse SSc (51), suggesting once more time his early implication in disease and highlighting a potential therapeutic window for the use of B-cell targeting therapies.

Proteomic analyses in early dcSSc patients showed a difference in the longitudinal course of serum proteins according to the ANA status (52), confirming a B-cell implication with a continuum of differential signature from the genetic level to the protein level.

## 5 Functional Characteristics

**Figure 3:**
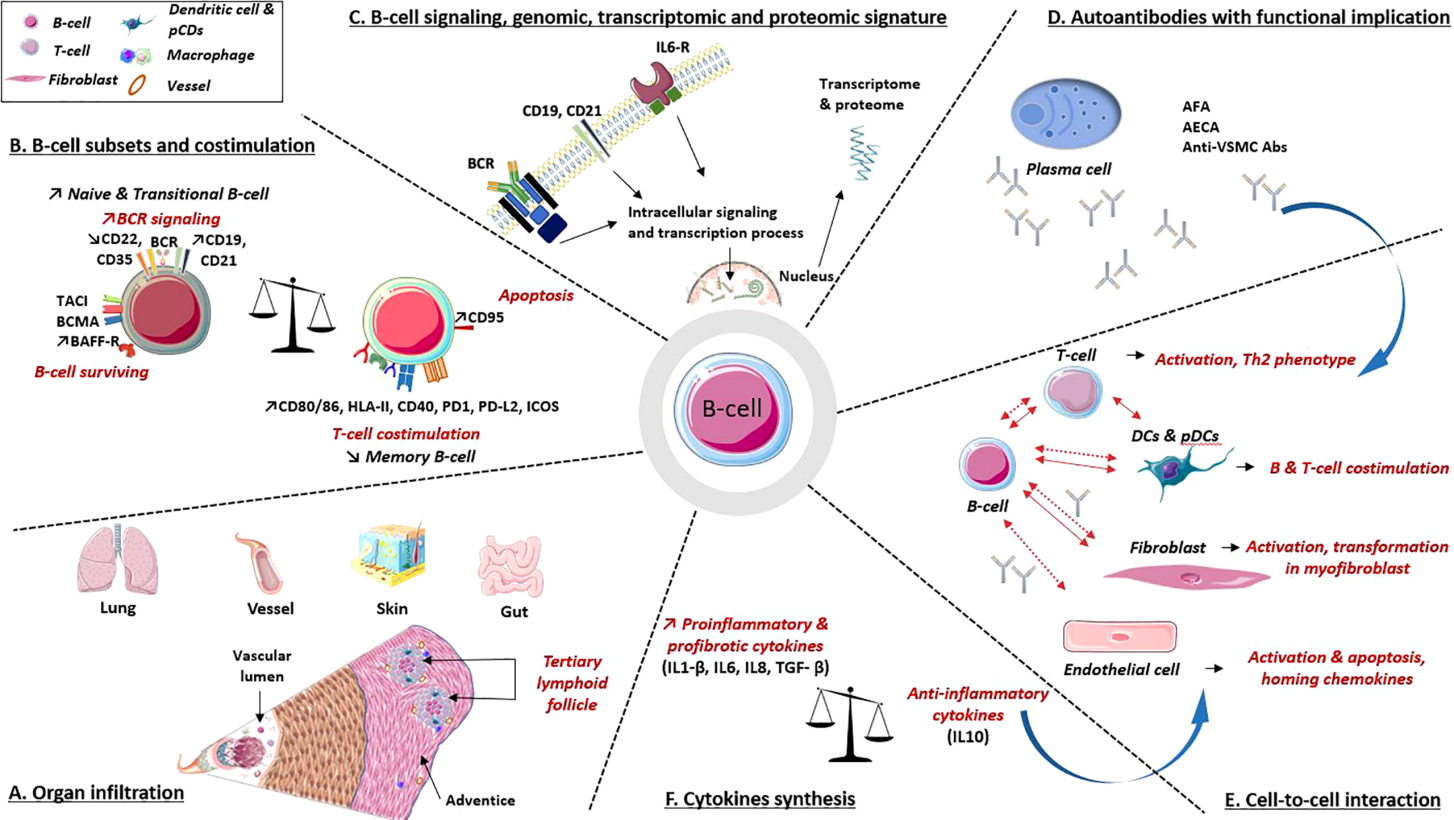
B-cell implications in the pathogenic processes involved in systemic sclerosis. **(A)** Organ infiltration: B-cell infiltrates are present in various organs involved in SSc. These infiltrates combine B and T cells, plasma cells, dendritic cells and macrophages, underlying their interactions. **(B)** B-cell subpopulations: B-cells express at their surfaces multiple molecules involved in activation or surviving pathway, as well as decreased levels of inhibitors coreceptors. B-cells also express co-receptors required for interaction with T cell. **(C)** B-cell intracellular signaling, genomic, transcriptomic and proteomic data argue a B-cell signature in SSc. **(D)** Autoantibodies. Several autoantibodies have demonstrated their involvement in cell activation, apoptosis induction, proinflammatory and profibrotic processes. **(E, F)** Cell to cell interactions and cytokines synthesis: B-cells interact with various immune cells, fibroblast and endothelial cell through direct (solid line) and indirect contact (i.e., cytokines and antibodies; dotted line). All participate in profibrotic, proinflammatory processes, vascular remodeling and immune dysregulations. Abs, antibodies; AECA, anti-endothelial cell Abs; AFA, anti-fibroblast Abs; anti-VSMC Abs, anti-vascular smooth muscle cell Abs; BAFF-R, calcium-modulator and cyclophilin ligand-interactor and BAFF receptor; BCMA, B-cell survival membrane receptors: B-cell maturation protein; BCR, B-cell receptor; CD, cluster of differentiation; DCs, dendritic cells; HLA-II, human leukocyte antigen class II; ICOS, inducible T-cell costimulatory; IL, interleukin; IL6-R, IL6 receptorPD-1/PD-L2: programmed death/programmed death ligand immune checkpoint; pDCs, plasmacytoid dendritic cells; TACI, trans-membrane activator.

B-cells contribute in many ways to the pathogenesis of SSc, namely, autoantibody synthesis, proinflammatory or profibrotic cytokine production, reduction of immunomodulatory cytokines, and/or through direct interactions with other cell types (**Figure 3**).

### 5.1 Autoantibodies Contribute to Cell Activation, Inflammation, Fibrosis, and Vasculopathy

The prevalence of anti-nuclear antibodies (Ab) in SSc patients is greater than 90% (53). Despite their important role in disease classification and prognosis staging, specific autoantibodies (mostly identified for centromeric proteins (CENP), topoisomerase I and RNA polymerase III) do not seem to play a major role in SSc pathogenesis (**Table 1**) (54, 69).

**Table 1:** Autoantibodies associated with Systemic Sclerosis.

Autoantibody target	Prevalence (%)	Clinical association	Vasculopathy	Fibrosis	Other	References
Centromere	20–40	lcSSc & PAH	−	−	–	(54)
Topoisomerase I	9–42	dcSSc & ILD	−	−	–	(54)
Fibrillarin (U3 RNP)	3–20	dcSSc	+	+	–	(54, 55)
RNA polymerase III	4–11	dcSSc, SRC & malignancy	−	−	–	(54)
Th/To	2–5	SRC	−	−	–	(54)
Ku	2–5	Overlap syndrome	−	−	–	(54)
Endothelial cell [anti-VCAM, ICAM-1, NAG-2]	22–85	PAH	+	−	Leukocyte adhesion	(56–58)
Angiotensin II type 1 Receptor (AT1R)	85	PAH & Digital ulcer	+	+	–	(56, 59)
Endothelin type A receptor (ETAR)	85	PAH ulcer	+	+	–	(56, 59)
M3R	65–80	Gastrointestinal involvement	−	−	Inhibition of type 3 muscarinic receptor	(60, 61)
Vascular smooth muscle cells	80	–	+	−	–	(56–58)
Fibroblast	25–60	dcSSc with PAH, ILD	−	+	Adhesive mechanism	(62–64)
MMP-1 & MMP-3	50	dcSSc	−	+	–	(65)
PDGF-Receptor	33	–	−	+	–	(66)
MSRA	33	ILD & cardiac involvement	+	+	Reinforcement of oxidative stress	(67)
PRX I	33	ILD & cardiac involvement	+	+	–	(67)
CD22	22	–	−	−	Antagonize inhibitory CD22 signal	(68)

Anti-AT1-Rc Abs, anti-angiotensin II type 1 receptor Abs; anti-ETa-Rc Abs, anti-endothelin 1 type A receptor Abs; anti-M3R Abs, anti-muscarinic acetylcholine receptor M3; anti-MSRA Abs, anti-Methionine Sulfoxide Reductase A Abs; anti-PDGF-Receptor Abs, anti-platelet-derived growth factor receptor Abs; anti-Prx I Abs, peroxiredoxin I; anti-RNA polymerase III Abs, anti-ribonucleid acid polymerase enzyme III Abs; anti-U3-RNP Abs, anti-U3 ribonucleoprotein Abs, anti-topoisomerase 1 Ab; Anti-VCAM Abs, anti-vascular cell adhesion molecules Abs; anti-VSMC Abs, anti-vascular smooth muscle cell Abs; CCL, chemokine ligand; CD, cluster of differentiation; CENP, centromeric protein with a molecular weight of 17 (CENP-A), 80 (CENP-B) and 140 kDa (CENP-C); dcSSc, diffuse cutaneous form of systemic sclerosis; ICAM, intercellular adhesion molecules; ILD, interstitial lung disease; Ku, p70/p80 is DNA-binding protein involved in DNA repair and regulation; lcSSc, limited cutaneous form of systemic sclerosis; MMP, matrix metalloproteinases; PAH, pulmonary arterial hypertension; SRC, scleroderma renal crisis; TM4SF7 or Nag-2, cell surface tetraspanin transmembrane 4 superfamily molecules member 7; Th/To, 7-2 RNP and 8-2 RNP that are subunits of human mitochondrial RNase and RNase P ribonucleoprotein complexes.

The anti-Topo I Ab is associated with the highest incidence of ILD and the poorest survival, followed by the anti-RNA polymerase III Ab. The anti-RNA polymerase III Ab is also associated with the highest incidence of scleroderma renal crisis (SRC). In contrast, SSc patients with an anti-U3 RNP Ab have the greatest incidence of pulmonary hypertension and SSc-related cardiac involvement (70, 71). Unbiased clustering methods incorporating autoantibody status also show their involvement in grading long-term disease prognosis (72).

Conversely, other autoantibodies, targeting cell surface receptors or ubiquitous molecules with potential direct pathogenic effects, have been identified in skin and pulmonary fibrosis, bowel involvement and/or vascular remodeling. The three main target cells are endothelial cells, fibroblasts, and vascular smooth muscle cells (VSMC). These autoantibodies induce activation and a proinflammatory or pro-adhesive phenotype of their cellular targets, and sometimes apoptosis (73). Thus, a direct pathogenic effect has been documented for certain anti-endothelial cells (AECA) and anti-fibroblast antibodies (AFA) (62–64, 74–76). This is the case for AECA targeting angiotensin II type I receptor or endothelin-1 type A receptor, which induce activation and apoptosis of endothelial cells with fibroblast activation, and secretion of profibrotic TGF-β cytokine (56, 77). Anti-platelet derived growth factor (PDGF)-receptor antibodies activate human VSMCs and fibroblasts and their transition into myofibroblasts, leading to fibrotic and vascular injury (66, 75). In the first publication, the presence of these anti-PDGF-receptor stimulatory antibodies was found in 100% of patients (n = 46). Their functional activity has been demonstrated through the involvement of the Ha-Ras-ERK1/2 activating cascade downstream of the PDFG receptor, the induction of ROS accumulation in a dose-dependent manner in *in vitro* experiments, and the promotion of type I collagen-related genes (66). Similar results have been demonstrated on human arterial smooth muscle cells (n = 11) with activation by anti-PDGF receptor antibodies of cell growth, migration ability, the generation of ROS, type I collagen gene expression, and the involvement of the NOX4 and mTORC1 pathways (75). These data must be relativized slightly because the sample sizes are low and, thus far, these results have not been replicated. Proteomic studies have identified numerous antigenic targets, such as Stress-Induced-Phosphoprotein 1 (STIP 1) or alpha-enolase, two proteins involved in the contraction of vascular smooth muscle cells, with an antibody-mediated direct pathogenic effect with contraction induced by incubation with purified IgG from SSc patients (78).

### 5.2 Imbalance in Proinflammatory, Profibrotic and Anti-Inflammatory Cytokines Produced by B-Cells in SSc

In SSc patients, activated B-cells secrete high levels of IL-6 and TGF-β. The IL-6 levels in the supernatant of purified B-cells from SSc patients are correlated with the mRSS, suggesting a pivotal role of B-cells in the fibrosis process (16). IL-6 stimulates B and T-cell proliferation, increases the resistance to apoptosis, activates fibroblasts and reduces the regulatory functions of Tregs. The IL-6 signaling induced indirectly the collagen synthesis by the fibroblast, through the phosphorylation of STAT3, the activation of downstream targeted genes, and *via* Gremlin-1, a *Bone Morphogenetic Protein* (BMP) antagonist, which activates the non-canonical TGF-β pathway *via smad3* (79). Furthermore, the highest serum levels of IL-6 are reported in the early dcSSc compared to the other groups of patients (80). Fibroblasts are also important producers of IL-6. Fibroblasts from affected skin areas in SSc patients produce significantly more IL-6 compared with fibroblasts from unaffected skin areas, or from healthy controls (81). There is, therefore, a loop between the fibroblast and the B-cell that is partly dependent on IL-6. Noteworthy, IL-6 and IL-10 serum levels correlated with skin and pulmonary fibrosis (82, 83). Otherwise, circulating B-cells secrete increased amounts of IL-8, IL-1β, BAFF, and CXCL13, which are proinflammatory, profibrotic cytokines or chemo-attractant molecules, both in scleroderma mouse models and SSc patients (20, 22, 37, 84). Many of these cytokines contribute to polarizing CD4^+^ T cells into IL-4 producing Th2 cells (85), T follicular helper (Tfh) and/or Th17 cells. In SSc, a Th2 lymphocyte phenotype is observed in the early stages of disease (86), and Th2 cytokines enhance antibody production by plasma cells and contribute to stimulating collagen production by fibroblasts.

Among regulatory cytokines, a particular interest has recently been given to IL-10, produced by Breg. Bregs can decrease inflammatory and autoimmune processes (87). These cells exert suppressive effects on a large variety of cells, including autoreactive T cells, dendritic cells, or macrophages through IL-10 production/synthesis. A reduction of IL-10 synthesis has been documented, both in mouse models and in SSc patients, and is correlated with the decrease of IL-10^+^ B-cells (19, 27, 84, 88). The decrease in these cells encountered in SSc patients is associated with the presence of ILD and inversely correlated with autoantibodies levels, and with Th17 T cells (26) suggesting their high implication in the control of the profibrotic environment (26).

### 5.3 B and T Cells Co-Interact and Self-Activate

The identification of B- and T-cell infiltrates in SSc patient skin biopsies led to arguments for their strong interaction (12, 89) and predominantly in the early stages of disease. As expected, the production of anti-topoisomerase I Ab requires direct contact and restriction to HLA-DR and CD40/CD40-ligand costimulatory molecules in collaboration with CD4^+^ T cells (90, 91). Follicular helper T cells (Tfhs) are crucial for the activation and differentiation of B-cells into plasma cells (92). Many data highlighted the interaction of Tfhs and B-cells through direct contact or *via* the intermediate of cytokines (IL-21) in the fibrotic process encountered in SSc patients or murine models (93, 94). Tfhs are hyperactivated in SSc patients through the higher expression of activation proteins like inducible T-cell costimulatory (ICOS) and PD-1 on their surface compared to healthy controls (93). Compared to healthy controls, circulating Tfh from SSc patients secreted higher IL-21 levels, promoting the differentiation of naïve B-cells into plasma cells and proliferation, and Ig secretion, especially in dcSSc and PAH (94).

B-cells interact with other T-cell subsets in SSc. The transmembrane glycoprotein T-cell Ig and Mucin domain protein 1 (TIM-1), a regulatory marker at the Breg surface, is implicated in the interaction between B and T cells in SSc. The frequency of peripheral blood TIM-1^+^ IL-10^+^ Breg is decreased in SSc patients compared with healthy controls, with a further defective ability to suppress CD4+ T-cell activation and inflammatory cytokine secretion (IFN-γ, TNF-α, IL-17) (27). However, the CD70 promoter is demethylated, favoring its overexpression on CD4+ T cells and seems to have contributed to the autoimmune response *via* its costimulatory activity of the B-cell during the B–T cell interaction (95). CD8^+^ effector T cells, Th17-cells and Treg are also involved in the synthesis of many proinflammatory and profibrotic cytokines in SSc (IL-4, IL-5, IL-9, IL-13, IL-17, TGF-β, and TNF-α) (96–98).

### 5.4 B-Cells Interact With Dendritic Cells and Other Innate Immune Cells

Very little data are available on the B-cell/dendritic cell (DC) interaction in the SSc. Activated B-cells induce the maturation of DCs in a contact-dependent manner (CD83, CD80, CD86, CD40, and HLA-DR) through BCR signaling and the BAFF receptor. B-cell-matured DCs secondary promote polarization and activation of naive CD4^+^ T cells into Th2 cells, producing increased amounts of IL-4, IL-5, and IL-13 (99).

Otherwise, proteome-wide analysis demonstrated a high level of CXCL4 in the blood and skin of SSc patients, and primarily synthesis by the plasmacytoid DCs (pDCs). CXCL4 levels were correlated with skin and lung fibrosis and with PAH (100). Similar data were shown in the BLM model with an increased number of pDCs in the affected skin and lungs compared to wild-type mice (101). The pDCs express the BAFF ligand (35), supporting the argument for an interaction between B-cells and pDCs in the SSc.

B-cells interact with other innate immune cells such as the macrophages. Granulocyte Macrophage-Colony Stimulating Factor (GM-CSF)^+^ B effector cells stimulate macrophages through GM-CSF synthesis to induce inflammatory and fibrotic lesions. These cells belong to the memory B-cell subset and strongly produce IL-6 and TNF-α. These cells have been reported to be increased in SSc patients, especially in dcSSc and associated with ILD, suggesting their role in the pathogenesis of SSc (102).

### 5.5 B-Cells Activate Fibroblasts

In cellular culture, cell-to-cell contact between B-cells and dermal fibroblasts induces an increase in collagen, α-smooth muscle actin, and metalloproteinase synthesis, or cytokines and chemokines (IL-6, TGF-β1, CCL2) (40, 41).

Antibody-mediated effects are illustrated with AFA, from the serum of SSc patients, which binds skin and lung fibroblasts, leading to proadhesive (ICAM-1), proinflammatory (reactive oxygen species (ROS)) and profibrotic (collagen) processes (66, 75, 103, 104) (**Table 1**).

Paracrine-mediated effects are illustrated by the ability of B-cell supernatant purified from SSc patients, rich in IL-6 and TGF-β, to induce fibroblast proliferation, collagen synthesis, and fibroblast-myofibroblast transition.

### 5.6 B-Cells Participate in Endothelial Cell Activation and Endothelial Damages

Interactions between B-cells, autoantibodies that they produce, and endothelial cells lead to their activation, immune cell homing, and apoptosis (56, 77). Purified IgG from AECA-positive SSc, PAH, or SLE patients is able to upregulate cell expression of various chemoattractant molecules (ICAM-1, VCAM-1, E-selectin, CCL2, or CXCL8), ROS production, interleukins (IL-6, -8) (56–58) and promotes intima hyperplasia and contributes to vascular injury (75).

## 6 B-cell as Therapeutic Target

Many data argue for the involvement of the B-cell in the early phase of SSc, or during some specific SSc-related involvement. Overall, the therapies directly targeting the B-cells, the cytokines produced by the B-cells during SSc, or these costimulation pathways therefore do not work in all patients, despite interesting effects (105). This suggests the potential interest of combined therapies, which have been developed in a few years. Over the past decade, interventional trials of biotherapies have been steadily increasing, representing about one-third of all therapeutic trials.

### 6.1 Depleting Therapies of B-Cell in SSc

#### 6.1.1 Direct Cytotoxicity of B-cell: The Anti-CD20 Monoclonal Antibody

B-cell depletion in SSc patients is associated with a decrease of the TGF-β pathway, the collagen and myofibroblast accumulation, and the attenuation of PDGFR expression and activation in the skin (106–108). In newborn TSK mice, B-cell depletion prevents skin fibrosis and autoantibody production (88).

Impact of anti-CD20 monoclonal antibody (rituximab RTX) on the course of SSc has been reported in two small randomized and controlled trials, respectively in 16 (versus placebo, ratio 1:1) and in 60 patients (versus cyclophosphamide, ratio 1:1) with early SSc (<2 years of duration) (109, 110). Despite the small number and the need for caution in interpretation, FVC improved slightly in the RTX group compared to the decrease in the cyclophosphamide group, as well as an improvement in mRSS in the RTX group (110). The same trends have been highlighted in RA-related ILD (111, 112). A recent systematic review with meta-analysis has assessed the impact of RTX on the changes in FVC and DLCO parameters in the SSc-ILD from 20 studies (2 RCT, 6 prospective studies, 5 retrospective studies, and 7 conference abstracts) representing 575 SSc patients. In summary, RTX slightly improves FVC and DLCO at 6 and 12 months of follow-up compared to baseline. In the 2 trials comparing RTX to another immunosuppressant, RTX was similar to controls on FVC and DLCO, but exposed to a lower risk of infection (113). Data from the RECITAL RCT (NCT01862926) evaluating RTX versus cyclophosphamide (CYC) in ILD related to SSc (N = 39, 38.6%), an idiopathic inflammatory myositis (N = 45, 44.6%), or mixed connective tissue disease (N = 17, 16.8%), were recently presented at the American Thoracic Society International Conference (ATS). Patients were randomized 1:1 to receive CYC (N = 50) or RTX (N = 51). Concerning the primary endpoint at 24 weeks, both RTX and CYC improved the FVC without the superiority of the RTX but with fewer adverse events and a reduction in corticosteroid exposure compared to CYC (114). Many trials are ongoing or published testing rituximab in SSc-related ILD alone, or in association with mycophenolate mofetil, MMF (EvER-ILD NCT02990286), or SSc-associated PAH (115). This suggests a potential place for rituximab in the therapeutic arsenal of early forms with skin or ILD manifestations. The results of larger randomized and controlled trials are awaited.

#### 6.1.2 Direct Cytotoxicity of B-Cell: The Anti-CD19 Monoclonal Antibody

A phase I, randomized, placebo-controlled trial testing inebilizumab, an anti-CD19 monoclonal antibody in 28 SSc patients (24 in inebilizumab and 4 in the placebo group), has shown a depletion of peripheral B and plasma cells, with a possible effect on skin fibrosis (mRSS), but 2 serious adverse effects in the inebilizumab group led to the discontinuation of the trial (116).

#### 6.1.3 Impact of Autologous Hematopoietic Stem Cell Transplantation (AHSCT) on B-Cell Homeostasis

Several data have shown an encouraging impact of AHSCT in patients with SSc (117–119). Post transplantation SSc patients have a reduced B-cell division rate, a restoration of the B-cell homeostasis, and a tolerant adaptative immune status. This is supported by post-transplant Breg levels rise, an increased Breg/memory B-cell ratio, with a long-term increase of IL10 production and recover their ability to suppress synthesis of Th1 cytokines by CD4^+^ T cells (120, 121). After transplant, the B-cell subsets show an increase of CD27/IgD^+^ naive B-cell percentage associated with a decrease of CD27^+^/IgD^+^ pre-switch memory, CD27^+^/IgD^−^ post-switched memory B-cells (122), extinction of interferon and inflammation transcriptomic molecular signatures (123), a decrease of IL-6 and TGF-β-producing B-cells (121).

### 6.2 Therapy Targeting Cytokines Produced by B-Cell

Serum IL-6 levels are increased in SSc patients, especially in cases of aggressive disease (16, 124). The neutralization of IL-6 by monoclonal antibodies or by immunization against a peptide from murine IL-6 in BLM mice has induced a reduction in dermal thickness (124). Additionally, the anti-IL-6 receptor monoclonal antibody (tocilizumab) induces a slightly reduction of collagen fiber bundles in the skin (125).

Based on these preliminary data, two multicenter, randomized, double-blind, placebo-controlled trials assessed the efficacy of tocilizumab on mRSS (primary endpoint) in SSc patients: the phase 2 faSScinate trial [n = 87 (126)] and the phase 3 focuSSced trial [n = 212 (127)]. Both of them failed on the primary endpoint, the difference in the mean change in mRSS. Secondary analysis showed a less consistent decline in predicted FVC in favor of the tocilizumab group. As expected, a shorter disease course and the presence of inflammatory biomarkers were associated with the best response. Its therapeutic place remains to be clarified, and further trials are necessary. The Food and Drug Administration (FDA) has just approved its use in SSc-ILD.

TGF-β appears as a central mediator in organ fibrosis and it is mostly produced by the B-cell in SSc. Its main target is the fibroblast (4). Data from SSc patients and TGF-β transgenic mice support the rationale for targeting TGF-β (128). Fresolimumab, a pan-specific anti-TGF-β antibody, has been tested in an open-label study on early and diffuse SSc patients. The expression of TGF-β-related genes in the skin of SSc patients decreases after treatment with fresolimumab, as with myofibroblast infiltration and the skin fibrosis (129). These are very preliminary results.

### 6.3 Therapy Targeting Cytokine Activating B-Cell: BAFF

Belimumab is a recombinant monoclonal antibody targeting soluble BAFF that promotes the B-cell apoptosis. In a TSK mouse model, BAFF antagonist inhibited the synthesis of autoantibodies and skin fibrosis (36). One double-blind randomized and controlled trial, assessed versus placebo in association with MMF, failed on significantly improving mRSS. It showed a decrease in genetic pathways, including BCR, TLR signaling, and profibrotic genes like TGF-β which were overexpressed in improved patients (130). Combined sequential therapy after a rituximab course is currently being studied in dcSSc patients (NCT03844061).

### 6.4 Proteasome Inhibitor Targeting Plasma Cell: The Bortezomib

Bortezomib, the first-in-class proteasome inhibitor, suppresses activated B-cells, plasma cells, and DC maturation and promotes immune-modulatory effects. This therapeutic class, used particularly in multiple myeloma, is very active and attractive. A human phase 2 trial is being recruited, testing a combination of bortezomib and MMF in SSc-related ILD patients (NCT02370693). In preliminary data on BLM, TSK/+, or Scl-cGVHD mouse models, bortezomib reduced skin infiltration of B-cells and the number of germinal centers, preventing dermal fibrosis with antifibrotic and anti-inflammatory efficacy as well vas a T-cell polarization toward a regulatory phenotype (104, 131, 132).

### 6.5 Others Drugs Targeting B-Cell Intracellular Signaling and Metabolism

Several other treatments are under study in SSc and can target B-cells. For example, some immunosuppressive therapies like pomalidomide are tested in SSc-related ILD in phase 2, randomized, placebo-controlled trials (133). Currently, no studies are underway regarding the assessment of SYK inhibitors in SSc patients. Encouraging studies have been conducted in several murine models (45, 46, 134).

## 7 Conclusion

Many advances have been made in understanding the involvement of B-cells in the pathogenesis of SSc. Besides autoantibody production, B-cells produce profibrotic and proinflammatory cytokines and interact by direct contact with the fibroblast or other immune cells. This is associated with a disorder of the homeostasis of B-cell subsets, membrane or intracellular signaling, arguing for its implication in the proinflammatory and profibrotic mechanisms. Many of these B-cell abnormalities encountered in SSc patients are associated with specific prognostic profiles. Treatments targeting B-cells, through their depletion by cytotoxicity, or the cytokines produced by the B-cells, or their costimulation molecules, seem interesting, probably in certain profiles of early patients with severe organic damage. This requires additional randomized controlled trials.

## Author Contributions

BT, BC, and LM have made contributions to conception and design of this review, drafting and revising it. All authors listed have made a substantial, direct, and intellectual contribution to the work and approved it for publication.

## Conflict of Interest

The authors declare that the research was conducted in the absence of any commercial or financial relationships that could be construed as a potential conflict of interest.

## Publisher’s Note

All claims expressed in this article are solely those of the authors and do not necessarily represent those of their affiliated organizations, or those of the publisher, the editors and the reviewers. Any product that may be evaluated in this article, or claim that may be made by its manufacturer, is not guaranteed or endorsed by the publisher.
